# Exosomes derived from microRNA-138-5p-overexpressing bone marrow-derived mesenchymal stem cells confer neuroprotection to astrocytes following ischemic stroke via inhibition of LCN2

**DOI:** 10.1186/s13036-019-0193-0

**Published:** 2019-08-28

**Authors:** Yiming Deng, Duanduan Chen, Feng Gao, Hong Lv, Guojun Zhang, Xuan Sun, Lian Liu, Dapeng Mo, Ning Ma, Ligang Song, Xiaochuan Huo, Tianyi Yan, Jingbo Zhang, Zhongrong Miao

**Affiliations:** 1 Department of Interventional Neuroradiology, Beijing Tiantan Hospital, Capital Medical University, No. 6, Tiantan Xili, Fengtai District, Beijing, 100050 People’s Republic of China; 2 China National Clinical Research Center for Neurological Diseases, Beijing, 100070 People’s Republic of China; 3 Center of Stroke, Beijing Institute for Brain Disorders, Beijing, 100069 China; 4 School of Life Science, Beijing Institute of Technology, Beijing, 100081 China; 5 Departments of Clinical Laboratory, Beijing Tiantan Hospital, Capital Medical University, Beijing, 100050 People’s Republic of China

**Keywords:** microRNA-138-5p, LCN2, Bone marrow-derived mesenchymal stem cells, Exosomes, Astrocytes, Ischemic stroke

## Abstract

**Background:**

MicroRNAs (miRNAs) are implicated in the progression of ischemic stroke (IS) and bone marrow-derived mesenchymal stem cells (BMSCs)-derived exosomes play a role in IS therapy. Herein we hypothesized that the BMSCs-derived exosomes containing overexpressed miR-138-5p could protect the astrocytes following IS involved with lipocalin 2 (LCN2).

**Methods:**

The differentially expressed gene related to IS was initially identified by bioinformatics analysis. miR-138-5p was predicted to regulate LCN2. The expression of miR-138-5p and LCN2 was altered in the oxygen-glucose deprivation (OGD)-induced astrocytes. Furthermore, the cell behaviors and inflammatory responses were evaluated both in astrocytes alone and astrocytes co-cultured with exosomes derived from BMSCs overexpressing miR-138-5p to explore the involvement of miR-138-5p and LCN2 in IS. Besides, middle cerebral artery occlusion (MCAO) mouse model was established to explore the effect of BMSCs-derived exosomal miR-138-5p in IS in vivo.

**Results:**

LCN2 was highly expressed in IS. Besides, LCN2 was a target gene of miR-138-5p. BMSCs-derived exosomes could be endocytosed by astrocytes via co-culture. Overexpression of miR-138-5p promoted the proliferation and inhibited apoptosis of astrocytes injured by OGD, accompanied by the reduced expression of inflammatory factors, which was achieved by down-regulating LCN2. More importantly, BMSCs delivered miR-138-5p to the astrocytes via exosomes and BMSCs-derived exosomal miR-138-5p alleviated neuron injury in IS mice.

**Conclusion:**

BMSCs-derived exosomal miR-138-5p reduces neurological impairment by promoting proliferation and inhibiting inflammatory responses of astrocytes following IS by targeting LCN2, which may provide a novel target for IS treatment.

## Introduction

Stroke remains the second leading contributor to the progressive increase observed in global deaths and disability. Ischemic stroke (IS) and hemorrhagic stroke are regarded as the two main types of stroke, among which IS is responsible for more than 80% of the total cases of stroke [1, 2]. In IS, astrocytes are capable of provision nutrients to neurons, regulating water, ion homeostasis and cerebral blood flow, maintaining the blood-brain barrier as well as mediating the extracellular level of glutamate, in addition to serving as a source of neuroprotectants [3]. Hence, it is essential to maintain astrocytes in normal physiological function. Inflammation is widely considered to exert a drastic effect on the pathogenesis of IS as it may lead to ischemic brain injury [4]. Astrocytic inflammation, a consequence of stroke, can aggravate ischemic lesions, while astrocytes can also be conducive for neuroprotection [5]. At present, several improvements in the arena of IS treatment have been achieved, including astrocyte-based therapy discovered as one of the promising therapies [6]. Notably, exosomes have been found as a promising endogenous drug delivery nanosystem for cerebral ischemia treatment [7]. Therefore, it is of significance to further explore the novel treatment of IS based on exosomes endocytosed by astrocytes.

Exosomes are identified extracellular lipid-structure vesicles that are 50 to 100 nm in diameter and can be secreted by all cell types, including bone marrow-derived mesenchymal stem cells (BMSCs) [8, 9]. Exosomes have been reported to ameliorate oxygen-glucose deprivation (OGD)-induced neuronal death, inflammatory responses as well as apoptotic signaling pathway changes [10]. The neutrophil gelatinase-associated lipocalin (LCN2) is an iron transport protein involved in brain injury [11]. LCN2 can be secreted by activated astrocytes to promote neuron death in response to neurodegeneration [12]. In addition, LCN2 is involved in inflammation caused by macrophages. Therefore, it is inferred intervention of LCN2 can be explored to alleviate neuron impairment. microRNAs (miRNAs), small noncoding RNAs being important for brain functions such as neural development and neurogenesis, are found to be involved in IS [13]. Notably, microRNA (miR)-138 has been reported to be capable of conferring protection against cerebral ischemia/reperfusion injury [14] and miR-138-5p exhibits differential expression in Alzheimer disease [15]. Bioinformatics prediction results revealed that LCN2 is a differentially expressed gene in IS and miR-138-5p is its regulatory gene, fueling our hypothesis that miR-138-5p and LCN2 play a role in the progression of IS. We subsequently asserted that BMSCs-derived miR-138-5p-overexpressing exosomes play a regulatory role in IS. Herein, this study was performed with the aim of exploring the effects associated with BMSCs-derived miR-138-5p-overexpressing exosomes on the proliferation and apoptosis of OGD-induced astrocytes as well as inflammation in IS, in connection with the regulation of LCN2.

## Materials and methods

### Ethics statement

The current study was conducted with the approval of the Ethics Committee of Beijing Tiantan Hospital, Capital Medical University under Approval number 201707003. All animal experimental procedures were performed in strict accordance with the Institutional Animal Care and Use Committee under Approval number CMU-IACUC-003.

### In silico prediction

Through the GEO database (https://www.ncbi.nlm.nih.gov/geo/), IS-related expression chips were obtained, and R-language “limma” package was used for differential analysis. |LogFC| > 2 and *p* value < 0.05 were applied as the screening criteria for differential genes. The “expression” package was utilized to construct a differential expression boxplot. The differentially expressed genes and miRNAs in IS were screened out from bioinformatics analysis and the data were analyzed from IS gene expression chips (GSE30655 and GSE35338) in the Gene Expression Omnibus (GEO) database. In an attempt to improve the neurological impairment caused by IS, we conducted upstream mechanism studies to predict the regulatory miRNAs targeting LCN2 and used jvenn (http://jvenn.toulouse.inra.fr/app/example.html) to compare prediction results of miRNAs and screen miRNAs.

### Primary astrocyte isolation and cell model establishment of OGD

The brain tissues of C57BL/6 neonatal mice (born within 24 h) were extracted. The meninges and blood vessels were then removed on ice. The cortical tissues were subsequently cut into pieces in serum-free Dulbecco’s modified Eagle’s Medium (DMEM)/F12 medium, detached with 0.125% trypsin at 37 °C for 2 min. The cells were subsequently suspended with DMEM/F12 medium containing 20% FBS. After 24 h, the medium was changed, with both the dead and non-adherent cells removed accordingly. After two passages, the cells were used for subsequent experimentation.

The purified cells were placed in a pre-set anaerobic incubator containing 5% ·CO_2_, 10% H_2_ and 85% N_2_. The cells were added to a sugar and serum free DMEM medium. After 4 h, the cells were extracted and used as an OGD model.

The astrocytes were permitted to grow along with the cell slide and then fixed with ice-methanol for 15 min. Next, 0.2% Triton X-100 was used to penetrate the membrane for 5 min. Membrane blockade was subsequently performed using 5% bovine serum albumin (BSA) for 1 h and incubated overnight with mouse anti-glial fibrillary acidic protein (GFAP) antibody (1: 200) at 4 °C. Fluorescein isothiocyanate (FITC)-labeled goat anti-mouse immunoglobulin G (IgG) antibody (1: 200) was incubated at room temperature for 1 h under conditions void of light. Afterwards, DAPI staining was performed on the nucleus for 8 min. The cells were then blocked with 50% glycerin and analyzed under a fluorescence microscope.

### Plasmid transfection

The oxygen-glucose deprived astrocytes at the logarithmic growth stage (4 × 10^5^ cells/well) were inoculated into a 6-well cell culture plate. When the cell confluency reached 80–90%, the cells were transfected based on the lipofectamine 2000 instructions (11668–019, Invitrogen, Carlsbad, California, USA). The plasmids of 100 pmol were diluted with 250 μL serum-free medium Opti-MEM (the final concentration added to cells was 50 nM), and 5 μL lipofectamine 2000 was diluted with 250 μL serum-free medium Opti-MEM. The aforementioned diluted products were mixed sufficiently and added to a 6-well plate 20 min later. After 48 h, the medium containing transfection solution in the wells was discarded and replaced with RPMI 1640 medium containing 10% FBS (Santa Cruz Biotechnology, Inc., Santa Cruz, CA, USA). The cells were then used for subsequent experimentation following incubation for 24 to 48 h,

Oxygen-glucose deprived astrocyte models with underexpressed and overexpressed miR-138-5p and LCN2 were constructed, after which the astrocytes were transfected with plasmids of miR-138-5p mimic negative control (NC), miR-138-5p inhibitor NC, miR-138-5p mimic, miR-138-5p inhibitor, shRNA (sh)-LCN2-NC, sh-LCN2, miR-138-5p mimic + sh-LCN2-NC and miR-138-5p mimic + sh-LCN2.

### Dual luciferase reporter gene assay

The targeting relationship between LCN2 and miR-138-5p were analyzed by biological prediction website https://cm.jefferson.edu/rna22/Precomputed and a dual luciferase reporter gene assay in order to verify the relationship. LCN2 dual luciferase reporter gene vector and mutant vector at the binding site with miR-138-5p, namely, PGLO-LCN2 wild type (WT) and PGLO-LCN2 mutant type (MUT), were subsequently constructed. The two reporter plasmids were then co-transfected into HEK293 T cells with miR-138-5p overexpression plasmid and NC plasmid, respectively. After 24 h of transfection, the cells were lysed and centrifuged for 1 min at 12000 rpm. Luciferase activity was detected by a Dual-Luciferase Reporter Assay System (E1910, Promega, Madison, WI, USA). Next, 100 μL fluorescein enzyme working fluid was added to each cell sample in order to detect the firefly luciferase activity; while the renilla luciferase activity was detected following the addition of 100 μL renilla luciferase working fluid. The ratio of firefly luciferase activity to Renilla luciferase activity was considered to be indicative of the relative luciferase activity. Each experiment was repeated three times.

Culture, isolation and characterization of BMSCs

The femur of the C57BL/6 mice was separated with the bilateral dry pulp ends then removed under sterile conditions. Sterile PBS was then used to repeatedly wash the bone marrow cavity, after which a sterile syringe was used to triturate the bone marrows that had been flushed out, ultimately permitting the bone marrow cells to be dispersed into the single cell suspension. Next, the bone marrow cells were collected into DMEM/F-12 medium containing 10% FBS and subsequently re-suspended. The cells were then inoculated into a culture flask at a density of 1 × 10^6^ cells/mL and cultured in a CO_2_ incubator. When the cell density reached approximately 80%, the cells were subcultured for BMSCs collection purposes [16].

### Lentivirus infection into BMSCs

The HEK-293 T cells were employed for transient co-transfection. PMD2G (1 μg), PSPAX2 (3 μg), and Prutou3-mChely/miR-138-5p (miR-NC/miR-138-5p) (4 μg) were mixed and used for lentivirus packaging. The collected supernatants were mixed in order to infect BMSCs. Next, a complex medium containing 500 μL lentivirus supernatant, 500 μL fresh medium and 8 μg polyacrylamide (Sigma-Aldrich Chemical Company, St Louis, MO, USA) was used to replace the former medium facilitating the uptake of virus particles. The BMSCs infected by lentivirus overexpressing miR-138-5p were used to co-culture with astrocytes.

### Isolation and characterization of exosomes

The BMSCs were inoculated into a 6-well plate at a density of 1 × 10^5^ cells per well. After overnight adherence, the exosome serum was replaced, followed by further culture for 48 h. A total of 5 mL supernatant was then collected from 3 duplicated wells, the exosomes (miR-138-5p) of which were extracted in strict accordance with the ExoQuick-TC™ kit (EXOTC10A-1, Shanghai Shanran Biotechnology Co., Ltd., Shanghai, China). The exosomes were identified by transmission electron microscopy (TEM).

Western blot analysis was used to detect the surface marker proteins CD63 and Hsp70 in exosomes. The expression levels of miR-138-5p among the exosomes were then detected by reverse transcription quantitative polymerase chain reaction (RT-qPCR). Each experiment was repeated three times.

### Acetylcholinesterase (AChE) activity detection

GW4869 inhibitor was employed to inhibit the secretion of BMSC-derived exosomes. The number of BMSC-derived exosomes was subsequently determined by AChE activity assay. Based on the operation instructions of the kit, the activity of AChE was determined using the 5,5′-dithiobis (2-nitrobenzoic acid) (DTNB) method, with the absorbance then measured at 412 nm by a microplate reader.

### Co-culture of astrocytes and BMSCs-derived exosome containing miR-138-5p

The 5-(and − 6)-carboxyfluorescein diacetate succinimidyl ester (CFSE) dye solution was diluted to 1: 1000 and added to 20 μg lentivirus-infected BMSCs suspension, and sufficiently mixing. After a period of rest at 37 °C for 15 min, PBS was added to the mixture and washed accordingly (100000×g, 70 min). Finally, CFSE-traced exosomes from BMSCs were co-cultured with astrocytes. After 24 h of co-culture, the uptake of exosomal miR-138-5p by astrocytes was analyzed under a confocal fluorescence microscope.

Fluorescence-labeled exosomes were co-incubated for 48 h with the supernatant of astrocytes that had been cultured in a 24-well plate with 50–60% confluency for 48 h for subsequent experiments. The astrocytes were treated respectively with DMSO, co-cultured with BMSCs and GW4869 (an inhibitor of exosome secretion), co-cultured with BMSCs-derived exosomes infected with miR-138-5p overexpression, co-cultured with BMSCs-derived exosomes infected with miR-NC, and co-cultured with BMSCs-derived exosomes without any treatment.

### RT-qPCR

The TRIzol kit (15596–018, Beijing Solarbio Science & Technology Co. Ltd., Beijing, China) was employed to extract total RNA in accordance with the provided instructions, followed by RNA concentration determination using Nanodrop. The primers used in this study were synthesized by Takara Biotechnology (Dalian, China) (Table 1). U6 and glyceraldehyde-3-phosphate dehydrogenase (GAPDH) were regarded as the internal reference primers, and the relative transcription level of the target gene was calculated using the 2^-△△Ct^ method: the fold changes were calculated by relative quantification [17]. Each experiment was repeated three times. The aforementioned procedures were also applicable for the animal experiments.

**Table 1:** Primer sequences for RT-qPCR

Gene	Sequences (5′-3′)	
miR-138-5p	F: GCCGGATAAGTGTTGTGGTCGA	R: ACTGAGCAAGCACTACCACCAGCA
LCN2	F: TGCCACTCCATCTTTCCTGTT	R: GGGAGTGCTGGCCAAATAAG
GAPDH	F: TTCACCACCATGGAGAAGGC	R: GGCATGGACTGTGGTCATGA
Caspase-3	F: TGTCATCTCGCTCTGGTACG	R: AAATGACCCCTTCATCACCA
Bax	F: TGCAGAGGATGATGCTGAC	R: CCTGTGACCTGAAGGAGG
Bcl-2	F: CCTGTGCCACCATGTGTCCATCC	R: GCTGAGAACAGGGTCTTCAGAGAC
U6	F: CTCGCTTCGGCAGCACA	R: AACGCTTCACGAATTTGCGT
IL-6	F: CCACTTCACAAGTCGGAGGCTT	R: CCAGCTTATCTGTTAGGAGA
IL-1β	F: GAAACAGCAATGGTCGGGAC	R: AAGACACGGGTTCCATGGTG
TNF-α	F: CGGAGTCCGGGCAGGT	R: GCTGGGTAGAGAATGGATGAACA

Note: RT-qPCR, reverse transcription quantitative polymerase chain reaction; F, forward; R, reverse; miR-138-5p, microRNA-138-5p; LCN2, lipocalin 2; GAPDH, glyceraldehyde-3-phosphate dehydrogenase; Bax, Bcl-2-associated X protein; Bcl-2, B-cell CLL/Lymphoma 2; IL-6, interleukin-6; IL-1β, interleukin-1β; TNF-α, tumor necrosis factor-α

### Western blot analysis

Tissue or cell total protein was extracted by RIPA lysate (R0010, Beijing Solarbio Science & Technology Co. Ltd., Beijing, China) in strict accordance with the provided instructions. The protein was separated by polyacrylamide gel electrophoresis (PAGE) and transferred onto polyvinylidene fluoride (PVDF) membranes via the wet transfer method. Next, membrane blockade was performed with 5% BSA, followed by overnight incubation at 4 °C (while shaking) along with the diluted antibodies: LCN2 (ab63929, 1: 500), CD63 (ab213090, 1: 500), Hsp70 (ab79852, 1: 1000), interleukin-6 (IL-6; ab193853, 1: 500), cleaved caspase-3 (ab13847, 1: 500), Bcl-2-associated X protein (Bax; ab32503, 1: 1000), B-cell CLL/Lymphoma 2 (Bcl-2; ab182858, 1: 2000). All the above antibodies were purchased from Abcam (Cambridge, MA, USA). The membranes incubated with peroxidase horseradish (HRP)-labeled goat-anti rabbit to IgG (ab205718, 1: 20000, Abcam, Cambridge, MA, USA) at room temperature for 1 h. The protein bands were visualized using the enhanced chemiluminescence (ECL) reagents for 3–5 min. The images were exposed on blue sensitive autoradiography film for 2 min. ImageJ 1.48u software (National Institutes of Health) was employed for protein quantitative analysis, with the ratio of the gray value of each protein to the gray value of the internal reference (GAPDH) considered as the relative protein expression. Each experiment was repeated three times. This procedure was also used in animal experiments.

### Detection of release rate of lactate dehydrogenase (LDH)

The culture medium from the culture dish was collected for evaluation purposes. The cells in the culture dish were scraped out using a cell scraper, and then moved to a 3 mL centrifuge tube. The cells were then centrifuged for 5 min at 1000 r/min in order to collect the supernatant. The LDH activity in the supernatant of the cell culture medium and cell homogenate was determined using the rate method, followed by calculation of the LDH release rate. LDH release rate = [LDH activity/(LDH activity of culture medium + LDH activity of cells)] × 100%. This procedure was also employed during the animal experiments. LIVE / DEAD staining was then performed in accordance with the kit instructions. The cells with green fluorescence were considered to be the living cells, while those with red fluorescence were regarded as the dead cells [18].

### Scratch test

The transfected cells were incubated for 24 h in an incubator at 37 °C with 5% CO_2_. The cells were transversely scratched on the monolayer cells with a 10 μL pipette gun. Cell migration was analyzed under an inverted microscope at 0 h and 24 h. The relative distances between the cells on both sides of the scratches were subsequently measured. The relative migration distance = distance difference / 2; relative cell migration rate = relative migration distance / distance from scratch edge to scratch center line at 0 h. Each experiment was repeated three times.

### 5-Ethynyl-2′-deoxyuridine (EdU) assay

The cells were cultured into the plates following a 48 h period of transfection. The cells were then subjected to incubation for 2 h along with 100 μL 50 μM EdU medium in each well. Afterwards, each well was added with 100 μL penetrant (0.5% Triton X-100) for 10-min incubation, with 100 μL 1 × Apollo staining solution for 30-min incubation, and with 100 μL Hoechst 33342 reaction solution for a 30-min incubation. The cell nuclei stained red were considered to be positive cells. The number of positive and negative cells was counted from three randomly selected visions under a microscope. The EdU labeling rate (%) = the number of positive cells/(the number of positive cells + the number of negative cells) × 100% [19]. Each experiment was repeated three times.

### Terminal deoxynucleotidyl transferase-mediated dUTP nick-end labeling (TUNEL) assay for astrocyte apoptosis

The 24-well plate was removed from the incubator and fixed with 4% polyformaldehyde solution. Next, 0.3% H_2_O_2_-formaldehyde solution was prepared for fixation. TUNEL reaction mixture was prepared using the TUNEL cell apoptosis detection kit (green fluorescence, C1088). The cells that had undergone varying treatments were then mixed with 50 μL TdT + 450 μL fluorescein-labeled dUTP solution, while the NC were only added with 50 μL fluorescein-labeled dUTP solution. The cells were later sealed using anti-fluorescence quenching blocking solution and subsequently observed under a fluorescence microscope at an excitation wavelength of 450 nm and an emission wavelength of 550 nm (green fluorescence).

### IS animal model establishment

Middle cerebral artery occlusion model (MCAO) was used to establish a cerebral ischemia-reperfusion model in mice [20]. Fifty C57BL/6 mice (8–10 weeks old) were purchased from the Experimental Animal Center and randomly selected for either the sham operation (*n* = 10) or the MCAO model establishment (the rest mice) based on body weight. The model establishment success rate was 75%. The mice were anesthetized using a mixture of oxygen and isoflurane (5% induction, 1.5% maintenance) and depilated with depilatory ointment (J. K. Nair & Company, Ahmadabad, India). The mice were fixed onto a heating pad in a supine position. The proximal end of the right common carotid artery and the carotid artery were ligated with a 6–0 polyester thread. MCAO was performed 10 mm distal to the bifurcation of the internal carotid artery, followed by ligation of the distal end of the internal carotid artery knot and fixation of thread embolus. Following induction of ischemia for 30 min, the thread embolus was removed and reperfusion began. After the conclusion of anesthesia, the mice were granted free access to food and water. The sham-operated animals subsequently underwent a median cervical incision, with the right common carotid artery separated and sutured directly, after which the mice were granted free access to food and water. The brain tissues of the experimental animals were collected for further experimentation.

### Animal grouping and treatment

With the exception of the sham group (mice that received sham operation), the other 30 successfully modeled mice were subsequently randomly assigned into three groups (*n* = 10 for each group): MCAO-control group (MCAO model mice without any treatment), MCAO + BMSCs-miR-NC group (MCAO model mice injected with NC exosomes) and MCAO + BMSCs-miR-138-5p group (MCAO model mice injected with BMSCs-miR-138-5p exosomes). Mice in each group were euthanized 4 weeks after treatment.

### Hematoxylin and eosin (HE) staining

The brain tissues of the mice were washed with 4 °C pre-cooled normal saline solution. The blood adhered to the surface was dried by filter paper, fixed by neutral formaldehyde, and paraffin sections were made. After baking at 60 °C for 1 h, the tissues were dewaxed with xylene and with gradient ethanol. Next, the tissues were stained with hematoxylin, and differentiated with 10% hydrochloric acid. The morphological changes in the brain tissue were then analyzed under a microscope (XSP-2C, Shanghai Bingyu Optical Instrument Co., Ltd., Shanghai, China).

### TUNEL assay for apoptosis of neurons

After dewaxing, the cells were sliced into 5 sections; each section was diluted with 1% protease K. Next, the sections were added with 0.3% H_2_O_2_ methanol solution, dripped with TUNEL reaction solution and incubated in a 37 °C humidity chamber for 1 h. When the cells exhibited brown-yellow nuclei under microscope investigation, the reaction was terminated with distilled water. Afterwards, the sections were counterstained with hematoxylin, and observed under an optical microscope (× 400), with 10 fields of vision selected from each section. The positive cells and podocytes were counted respectively. The cells displaying brown-yellowish nuclei were regarded as positive apoptotic cells while the cells with blue nuclei were considered to be normal cells. The average number of positive apoptotic cells as well as the normal cells was subsequently calculated. The apoptotic index (AI) was reflected by the ratio of the number of positive apoptotic cells to that of the normal cells.

### 2,3,5-triphenyltetrazolium chloride (TTC) staining

The brain tissues were promptly cut along the coronal plane. The thickness of each piece of brain tissue was confirmed to be approximately 2 mm, after which TTC staining solution was prepared. The tissue sections were stained with TTC for 30 min in a water bath under conditions void of light, at a constant temperature of 37 °C. After staining, the tissue sections were fixed in 4% paraformaldehyde for 24 h. The brain tissues stained red were representative of the normal brain tissues, while the unstained tissues were regarded as the infarcted regions. The infarct volume was calculated based on the section thickness and infarct area of each section.

### Nissl staining

Following ischemia and reperfusion, the paraffin sections were baked at 55 °C for 30 min, dewaxed with xylene and, immersed in gradient ethanol. According to the instructions of the Nissl staining kit, Nissl dye solution was added to the sections, which were then immersed into a 58 °C water bath for 40 min and promptly differentiated in the differentiation solution. After dehydration with 100% ethanol, the sections were cleared with xylene. The cleared sections were then finally sealed with neutral gum, and finally observed and photographed under a microscope.

### Statistical analysis

The SPSS 21.0 software (IBM Corp., Armonk, NY, USA) was employed for data analysis. The measurement data were presented as mean ± standard deviation. All data were processed with an evaluation of normality using D’Agostino & Pearson omnibus normality test. In regard to data displaying normal distribution, an independent sample *t* test was applied for the comparison between two groups and one-way analysis of variance (ANOVA) for comparison among multiple groups. In the event the data failed to exhibit normal distribution, a Kruskal-Wallis with Dunn’s multiple comparison post-hoc test was adopted for data analysis, followed by Pearson correlation analysis. A *p* < 0.05 value was considered to be indicative of statistical significance.

## Results

### LCN2 is highly expressed in IS and may affect the OGD-induced astrocytes

**Fig. 1:**
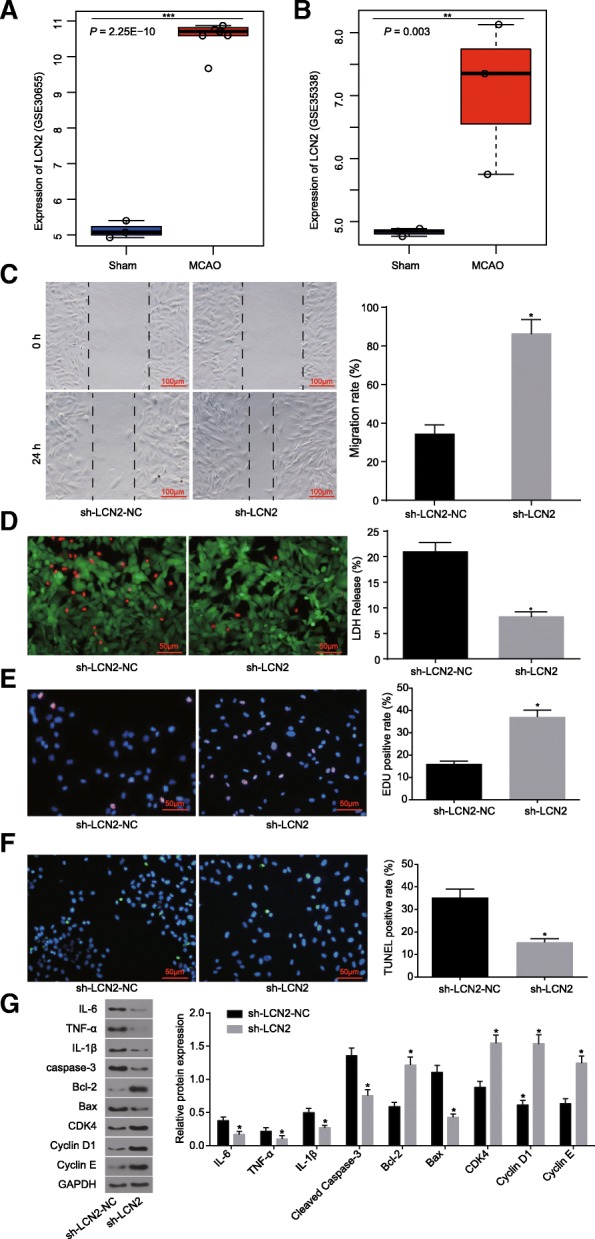
LCN2 is highly expressed in IS and may affect the proliferation and apoptosis of OGD-induced astrocytes. **a** the expression of LCN2 in the GSE30655 chip; *** *p* < 0.001. **b**, the expression of LCN2 in the GSE35338 chip; ** *p* < 0.01. **c**, the ability of cell repair determined by scratch test (scale bar = 100 μm); **d**, LIVE/DEAD staining (scale bar = 50 μm) and quantitative analysis for LDH release rate; **e**, cell proliferation detected by EdU staining (scale bar = 50 μm); **f**, TUNEL staining for astrocyte apoptosis (scale bar = 50 μm); **g**, Western blot analysis for protein bands and levels of inflammatory factors, proliferation and apoptosis marker proteins. The data were all expressed as mean ± standard deviation. The comparison between two groups was analyzed by non-paired *t*-test. The experiment was repeated three times. *, *p* < 0.05 vs. sh-LCN2-NC. IS, ischemic stroke; MCAO, middle cerebral artery occlusion model; GAPDH, glyceraldehyde-3-phosphate dehydrogenase; Bax, Bcl-2-associated X protein; Bcl-2, B-cell CLL/Lymphoma 2; IL-6, interleukin-6; IL-1β, interleukin-1β; TNF-α, tumor necrosis factor-α; sh-LCN2-NC, astrocytes transfected with sh-LCN2-NC plasmid; sh-LCN2, astrocytes transfected with sh-LCN2

Initially, the IS-related expression profiles GSE30655 and GSE35338 were retrieved from the GEO database. The LCN2 gene was found to be highly expressed in IS mice (Fig. 1a and b). The differential expression of LCN2 in tissues of the IS mice and sham-operated mice was subsequently evaluated. The previous results showed that astrocytes secreted LCN2 to promote neuroinflammation and play an important role in IS progression [21].

In an attempt to elucidate the regulatory effect of LCN2 on astrocytes under OGD conditions, the oxygen-glucose deprived astrocytes were treated with sh-LCN2-NC and sh-LCN2. The results obtained indicated astrocytes treated with sh-LCN2 exhibited an increased migration rate (Fig. 1c), lower LDH release rate (Fig. 1d), stimulated migration (Fig. 1e) and relatively lower apoptotic rate (Fig. 1f) than those treated with sh-LCN2-NC (*p* < 0.05). Next, Western blot analysis was performed in order to determine the expression levels of inflammatory factors, as well as proliferation and apoptosis marker proteins. The results revealed that sh-LCN2 contributed to the marked reduction in the expression of inflammatory factors, including IL-6, IL-1β, TNF-α, cleaved caspase-3 and Bax and increased Bcl-2 expression and significantly elevated expression of cell cycle markers, including CDK-4, Cyclin D1 and Cyclin E (*p* < 0.05) (Fig. 1g). The above results suggest that inhibiting the expression of LCN2 gene could promote the proliferation of OGD-induced astrocytes and inhibit the expression of both inflammatory and pro-apoptotic factors.

### LCN2 is a target gene of miR-138-5p

**Fig. 2:**
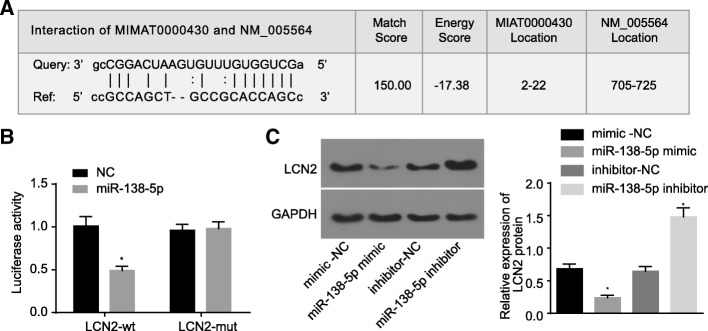
LCN2 is a target gene of miR-138-5p. **a**, predicted binding sites of miR-138-5p in 3’UTR of LCN2; **b**, luciferase activity of HEK293 cells detected by dual luciferase reporter gene assay; **c**, protein bands and quantitative analysis for LCN2 expression in astrocytes. The comparison among multiple groups was analyzed by one-way analysis of variance and the comparison between two groups was analyzed by non-paired *t*-test. The experiment was repeated three times. *, *p* < 0.05 vs. NC and mimic-NC. NC, negative control; WT, wide type; MUT, mutant type; GAPDH, glyceraldehyde-3-phosphate dehydrogenase; mimic-NC, astrocytes transfected with mimic-NC; miR-138-5p mimic, astrocytes transfected with miR-138-5p mimic; inhibitor-NC, astrocytes transfected with inhibitor-NC; miR-138-5p inhibitor, astrocytes transfected with miR-138-5p inhibitor

In order to alleviate the neurological impairment associated with IS, we investigated the upstream mechanism involving LCN2. Based on RAID v2.0 (http://www.rna-society.org/raid/index.html), subsequently predicting the existence of binding sites of miR-138-5p in LCN2 mRNA. LCN2 was identified as the target gene of miR-138-5p (Fig. 2a). Dual luciferase reporter gene assay was employed to verify the target gene of LCN2 (Fig. 2b). The results revealed that compared with NC group, the luciferase activity in WT 3’UTR of LCN2 was significantly inhibited by miR-138-5p (*p* < 0.05), while that in MUT 3’UTR was not significantly different (*p* > 0.05). The expression of LCN2 protein was subsequently detected by Western blot analysis (Fig. 2c), suggesting that the treatment of miR-138-5p mimic brought about a significant decrease in the expression of LCN2 protein (*p* < 0.05), while the miR-138-5p inhibitor led to a marked increase in the expression of LCN2 protein (*p* < 0.05). The aforementioned results suggested that miR-138-5p could specifically bind to 3′-UTR of LCN2 and downregulate the gene expression of LCN2 at the post-transcriptional level.

### Overexpression of miR-138-5p inhibits apoptosis of astrocytes injured by OGD

**Fig. 3:**
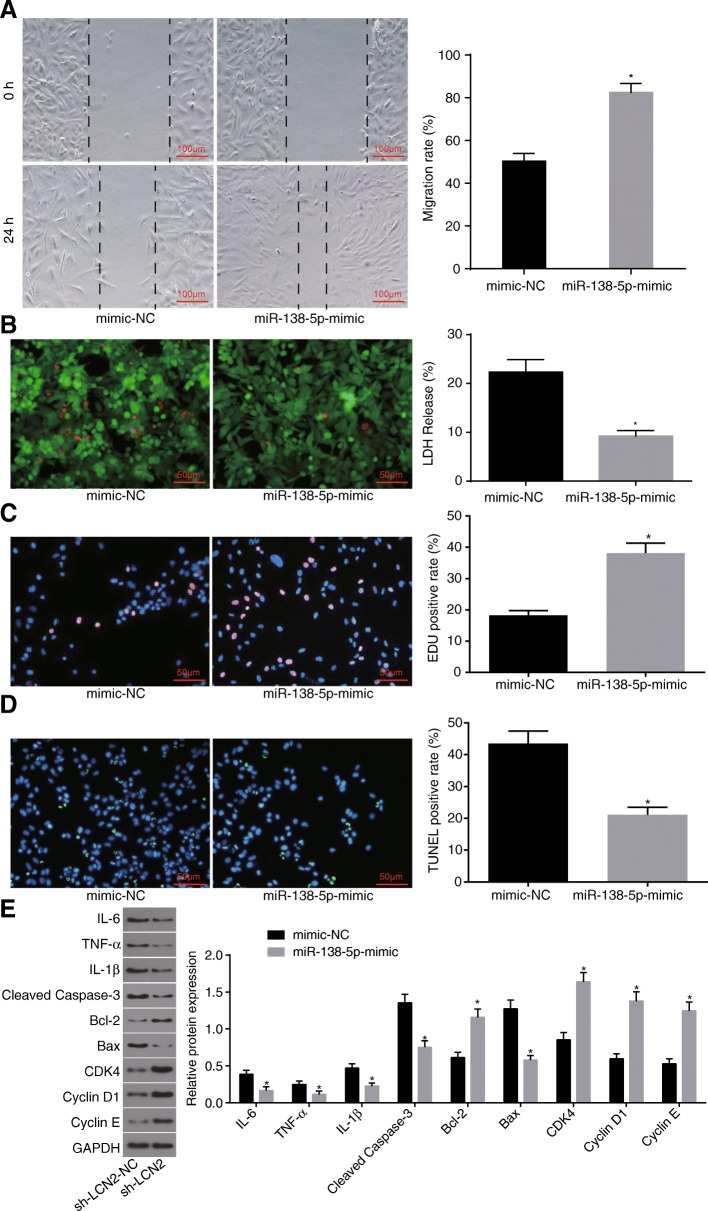
Overexpression of miR-138-5p inhibits apoptosis of astrocytes injured by OGD. **a**, the repair ability of damages in cells determined by scratch test (scale bar = 100 μm), and quantitative analysis for cell migration and the number of exosomes; **b**, LIVE / DEAD staining for LDH release rate (scale bar = 50 μm); **c**, cell proliferation detected by EdU staining and quantitative analysis for EdU positive expression (scale bar = 50 μm); **d**, TUNEL staining (scale bar = 50 μm) and quantitative analysis for TUNEL positive rate; **e**, protein bands and quantitative analysis for inflammatory factors, proliferation and apoptosis marker proteins determined by Western blot analysis. The data were all expressed as mean ± standard deviation. The comparison between two groups was analyzed by non-paired *t*-test. The experiment was repeated three times. *, *p* < 0.05 vs. mimic-NC. OGD, oxygen-glucose deprivation; LDH, lactate dehydrogenase; EdU, 5-Ethynyl-2′-deoxyuridine; TUNEL, terminal deoxynucleotidyl transferase-mediated dUTP nick-end labeling; GAPDH, glyceraldehyde-3-phosphate dehydrogenase; Bax, Bcl-2-associated X protein; Bcl-2, B-cell CLL/Lymphoma 2; IL-6, interleukin-6; IL-1β, interleukin-1β; TNF-α, tumor necrosis factor-α; mimic-NC, astrocytes transfected with mimic-NC plasmid; miR-138-5p-mimic, astrocytes transfected with miR-138-5p-mimic plasmid

In order to elucidate the effect associated with the overexpression of miR-138-5p on the astrocytes injured by OGD, the cultured astrocytes were treated with inhibitor-NC and miR-138-5p inhibitor. The results indicated that there to be a higher astrocyte migration rate following treatment with miR-138-5p mimic more so than in treatment with mimic-NC (*p* < 0.05) (Fig. 3a). Next, LIVE / DEAD staining revealed that miR-138-5p mimic resulted in few cells in loose cell structure, swelling, rupture or dissolution in different degrees, significantly reduced red-stained nucleus, lower LDH release rate (Fig. 3b), stimulated proliferation (Fig. 3c) and a reduced apoptotic rate (Fig. 3d). Western blot analysis was performed in order to determine protein levels of inflammatory factors, as well as proliferation and apoptosis marker proteins. The expressions of IL-6, IL-1β, TNF-α, cleaved caspase-3 and Bax exhibited a marked decline after treatment with miR-138-5p mimic accompanied by elevated expression of Bcl-2, while that of CDK-4, Cyclin D1 and Cyclin E exhibited a increase (*p* < 0.05) (Fig. 3e). These results demonstrated that overexpression of miR-138-5p promotes the proliferation and migration of astrocytes injured by OGD, and inhibits apoptosis.

### Downregulation of miR-138-5p promotes apoptosis of astrocytes injured by OGD

**Fig. 4:**
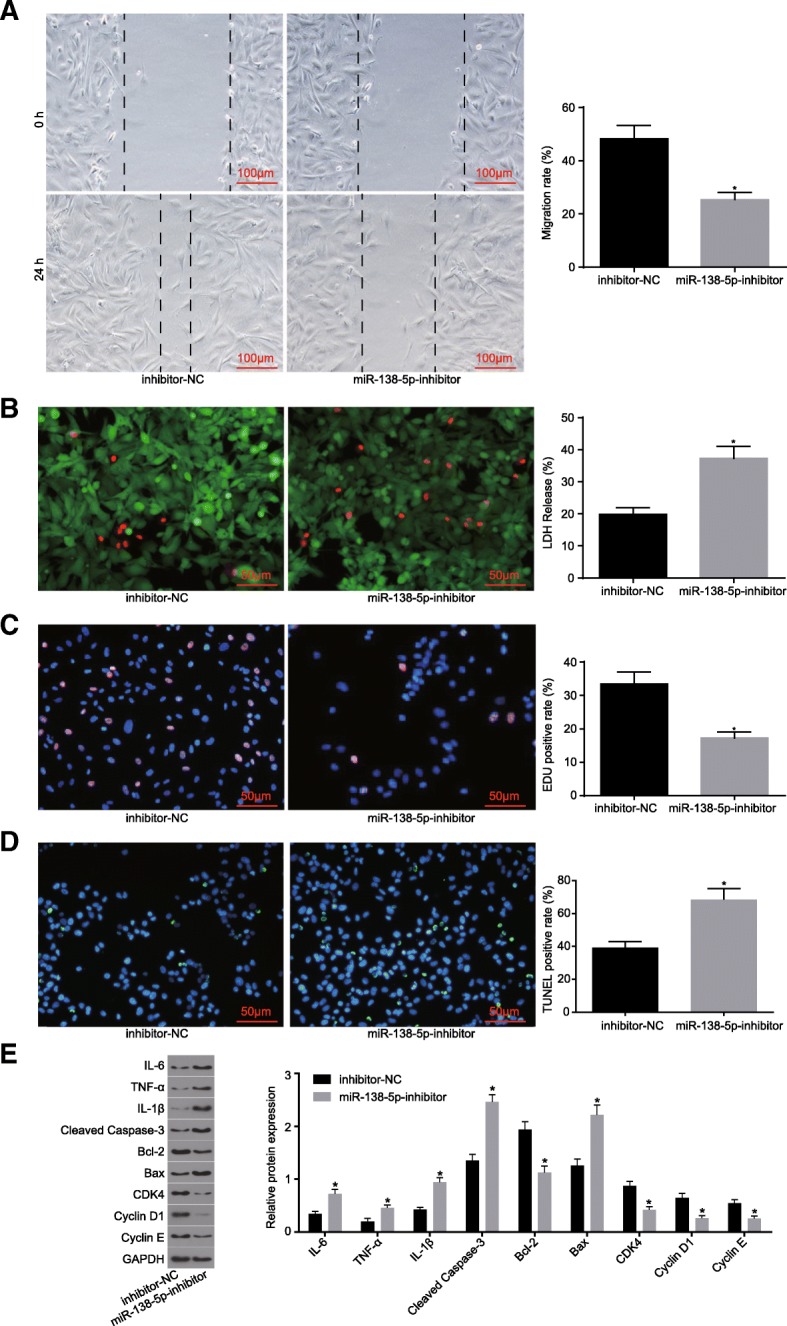
Downregulation of miR-138-5p promotes apoptosis of astrocytes injured by OGD. **a**, the repair ability of damages in cells determined by scratch test (scale bar = 100 μm), and quantitative analysis for cell migration and the number of exosomes; **b**, LIVE / DEAD staining for LDH release rate (scale bar = 50 μm); **c**, cell proliferation detected by EdU staining and quantitative analysis for EDU positive expression (scale bar = 50 μm); **d**, TUNEL staining (scale bar = 50 μm) and quantitative analysis for TUNEL positive rate; **e**, expression of inflammatory factors, proliferation and apoptosis marker proteins determined by Western blot analysis. The data were all expressed as mean ± standard deviation. The comparison between two groups was analyzed by non-paired *t*-test. The experiment was repeated three times. *, *p* < 0.05 vs. inhibitor-NC. OGD, oxygen-glucose deprivation; LDH, lactate dehydrogenase; EdU, 5-Ethynyl-2′-deoxyuridine; TUNEL, terminal deoxynucleotidyl transferase-mediated dUTP nick-end labeling; GAPDH, glyceraldehyde-3-phosphate dehydrogenase; Bax, Bcl-2-associated X protein; Bcl-2, B-cell CLL/Lymphoma 2; IL-6, interleukin-6; IL-1β, interleukin-1β; TNF-α, tumor necrosis factor-α; inhibitor-NC, astrocytes transfected with inhibitor-NC plasmid; miR-138-5p-inhibitor, astrocytes transfected with miR-138-5p-inhibitor plasmid

In order to investigate the role by which interfering with the expression of miR-138-5p exerts on astrocytes injured by OGD, the cultured astrocytes were treated with inhibitor-NC and miR-138-5p inhibitor. The results suggested there was a lower astrocyte migration rate following treatment with miR-138-5p inhibitor when compared to the results following treatment with inhibitor-NC (*p* < 0.05) (Fig. 4A). The results indicated there was a loose structure, swelling, rupture or dissolution at varying degrees, accompanied with elevated red-stained nucleus, higher LDH release rate (Fig. 4b), reduced proliferation (Fig. 4c) and increased apoptotic rate among cells treated with miR-138-5p inhibitor (Fig. 4d). Inflammatory factors, as well as proliferation and apoptosis marker proteins were through the application of a Western blot analysis, the results of which revealed that the expression of IL-6, IL-1β, TNF-α, cleaved caspase-3 and Bax was increased, which was accompanied by reduced Bcl-2, after treatment with miR-138-5p inhibitor, while that of CDK-4, CyclinD1 and Cyclin E displayed a decline (*p* < 0.05) (Fig. 4e). The aforementioned results suggest that inhibition of miR-138-5p suppresses the proliferation and migration of astrocytes injured by OGD, while promoting cell apoptosis.

### miR-138-5p exerts effects on astrocytes by regulating LCN2 gene

**Fig. 5:**
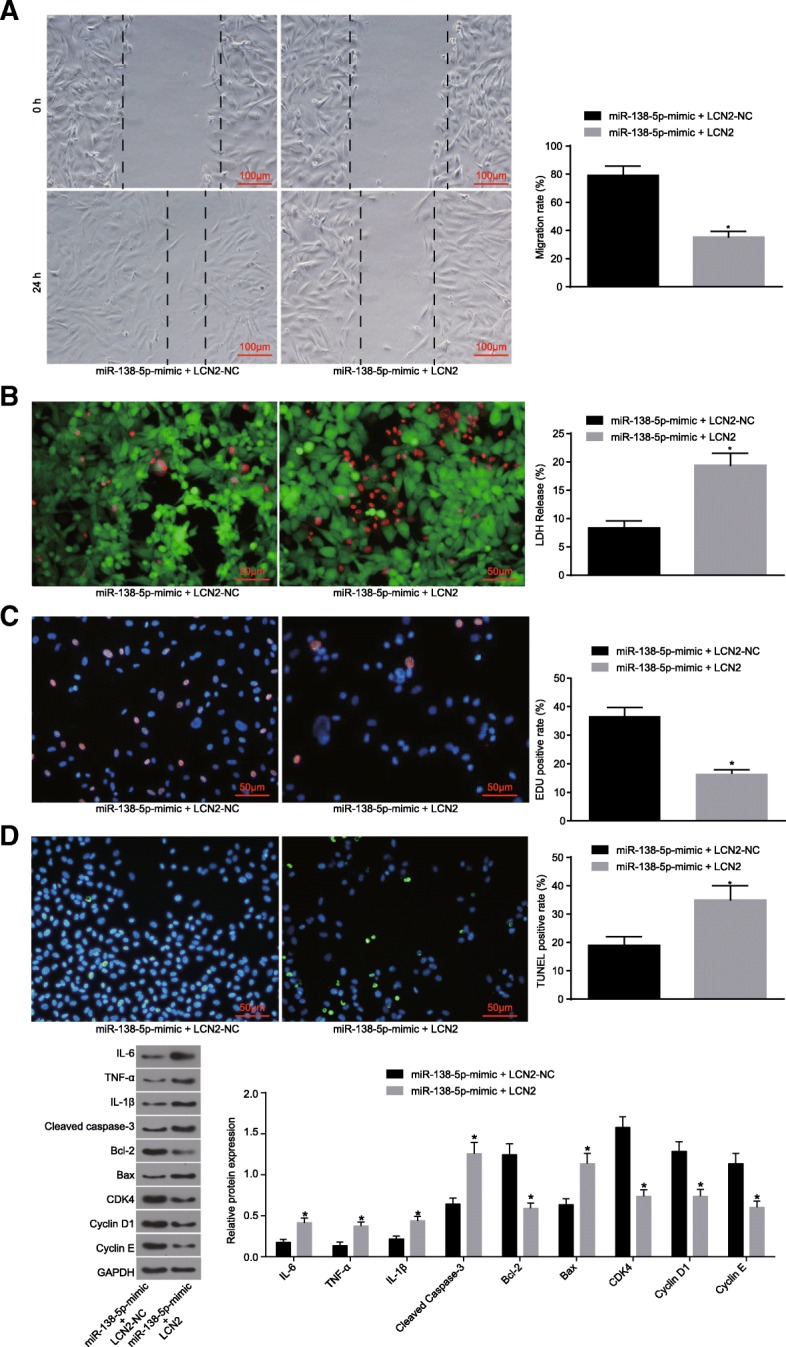
miR-138-5p plays a role in proliferation, apoptosis and inflammation of astrocytes by regulating LCN2 gene. **a**, the repair ability of damages in cells determined by scratch test (scale bar = 100 μm), and quantitative analysis for cell migration and the number of exosomes; **b**, LIVE/DEAD staining for LDH release rate (scale bar = 50 μm); **b**, cell proliferation detected by EdU staining and quantitative analysis for EdU positive expression (scale bar = 50 μm); **d**, TUNEL staining (scale bar = 50 μm) and quantitative analysis for TUNEL positive rate; **e**, expression of inflammatory factors, proliferation and apoptosis marker proteins determined by Western blot analysis. The data were all expressed as mean ± standard deviation. The comparison between two groups was analyzed by non-paired *t*-test. The experiment was repeated three times. *, *p* < 0.05 vs. miR-138-5p -mimic + LCN2-NC. OGD, oxygen-glucose deprivation; LDH, lactate dehydrogenase; EdU, 5-Ethynyl-2′-deoxyuridine; TUNEL, terminal deoxynucleotidyl transferase-mediated dUTP nick-end labeling; GAPDH, glyceraldehyde-3-phosphate dehydrogenase; Bax, Bcl-2-associated X protein; Bcl-2, B-cell CLL/Lymphoma 2; IL-6, interleukin-6; IL-1β, interleukin-1β; TNF-α, tumor necrosis factor-α; miR-138-5p-mimic + LCN2-NC, astrocytes transfected with miR-138-5p-mimic + LCN2-NC plasmid; miR-138-5p-mimic + LCN2, astrocytes transfected with miR-138-5p-mimic + LCN2

To study the regulatory effect of miR-138-5p on LCN2 expression in astrocytes, repair experiments were conducted with the treatment of miR-138-5p-mimic + LCN2-NC and miR-138-5p-mimic + LCN2. The ability of cell repair was assessed by scratch test. Relative to the control group, miR-138-5p-mimic with LCN2 overexpression was found to significantly inhibit cell migration (*p* < 0.05) (Fig. 5a). LIVE / DEAD staining demonstrated that the treatment of miR-138-5p-mimic + LCN2 resulted in a loose cell structure, swelling, rupture or dissolution, red-stained nuclei, in addition to higher LDH release rate (Fig. 5b), significantly decreased cell proliferation (*p* < 0.05) (Fig. 5c) as well as a relatively greater apoptotic rate (Fig. 5d). Western blot analysis revealed that under the effect of miR-138-5p + LCN2, the expression of IL-6, IL-1β, TNF-α, cleaved caspase-3 and Bax was significantly increased, which was accompanied by a reduction in the expression of Bcl-2 (*p* < 0.05) and the cell cycle factors CDK-4, CyclinD 1 and Cyclin E (*p* < 0.05) (Fig. 5e). These results demonstrated that miR-138-5p promotes the proliferation of astrocytes and reduces the expression of inflammatory and pro-apoptosis factors by regulating the LCN2 gene.

### Endocytosis of BMSCs-derived exosomes by astrocytes

**Fig. 6:**
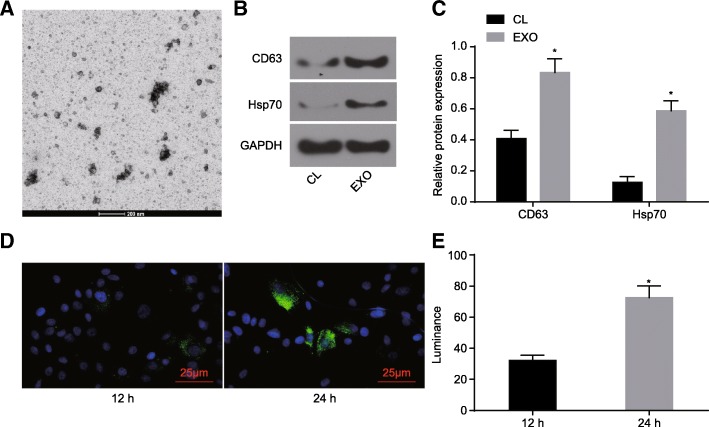
Endocytosis, intracellular sorting, and processing of BMSCs-derived exosomes by astrocytes. **a**, electron microscopic examination of exosomes (scale bar = 200 nm); **b**, protein bands of surface makers of BMSCs-derived exosomes determined by Western blot analysis (CL, BMSC lysate; EXO, exosomes); **c**, quantitative analysis regarding protein expression of surface makers of BMSCs-derived exosomes determined by Western blot analysis; **d**, exosome surface fluorescence (scale bar = 25 μm); **e**, quantitative analysis for endocytosis at different time points. The data were all expressed as mean ± standard deviation. The comparison between two groups was analyzed by non-paired *t*-test. The experiment was repeated three times. *, *p* < 0.05 vs. CL or endocytosis at 12 h. BMSCs, bone marrow-derived mesenchymal stem cells

BMSCs-derived exosomes were analyzed under a TEM (Fig. 6a). The expression of the exosomal surface markers CD63 and Hsp70 was identified by Western blot analysis both in the BMSC lysates and in the exosomes (Fig. 6b-c). Analysis of the fluorescence labeling of astrocyte-secreted exosomes under a microscope, revealed the fluorescence brightness increased significantly with time (Fig. 6d-e).

### BMSCs deliver miR-138-5p to the astrocytes via the exosomes

**Fig. 7:**
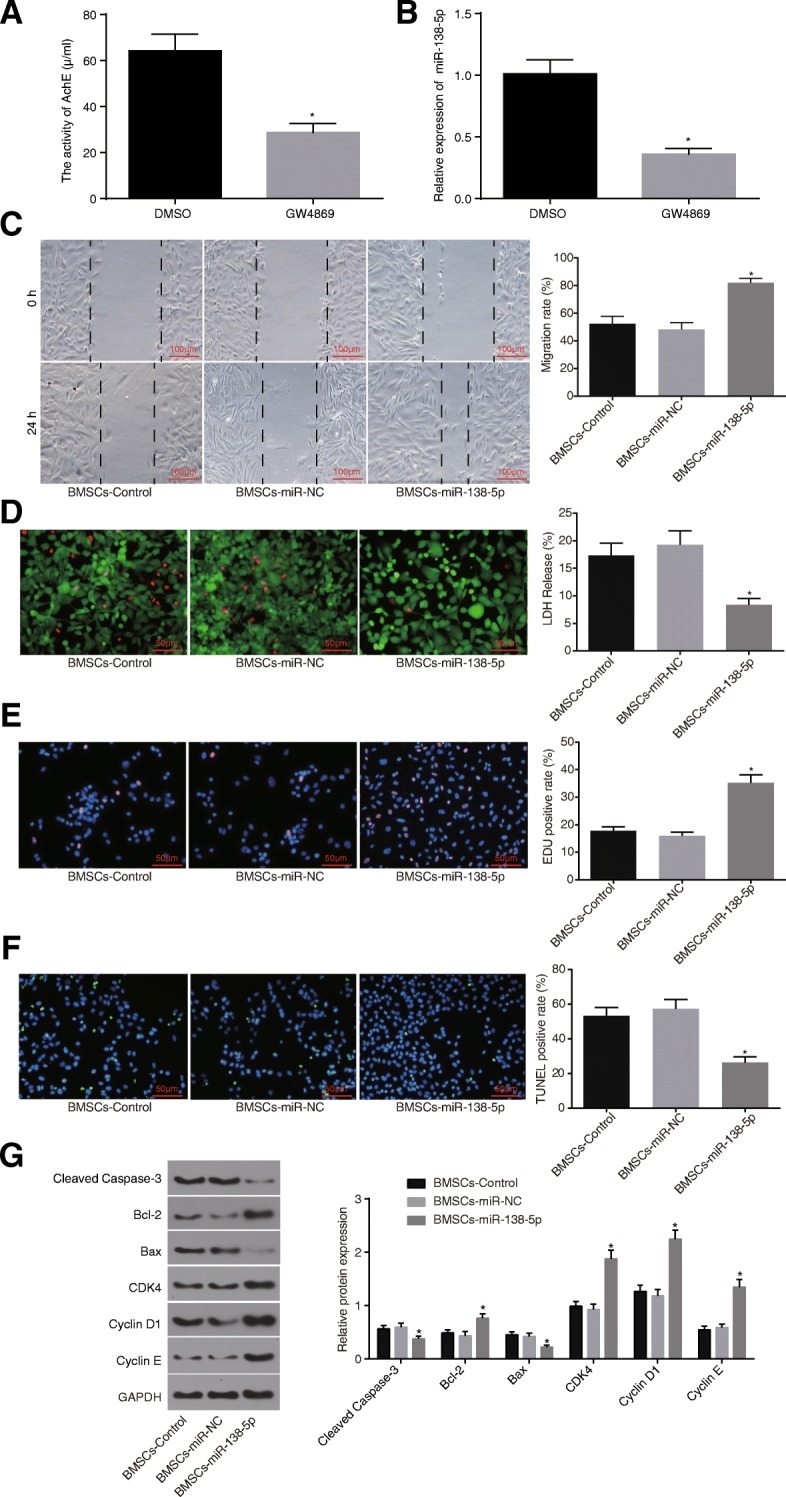
BMSCs deliver miR-138-5p to the astrocytes via the exosomes. **a**, quantitative analysis for the number of exosomes; **b**, the expression of the miR-138-5p in the co-culture system detected by RT-qPCR; **c**, repair ability of damages in cells determined by scratch test (scale bar = 100 μm), and quantitative analysis for cell migration and the number of exosomes; **d**, LIVE / DEAD staining for LDH release rate (scale bar = 50 μm); **e**, cell proliferation detected by EdU staining and quantitative analysis for EDU positive expression (scale bar = 50 μm); **f**, TUNEL staining (scale bar = 50 μm) and quantitative analysis for TUNEL positive rate; **g**, protein bands and quantitative analysis for expression of inflammatory factors, proliferation and apoptosis marker proteins determined by Western blot analysis. The data were all expressed as mean ± standard deviation. The comparison among multiple groups was analyzed by one-way analysis of variance. The experiment was repeated three times. *, *p* < 0.05 vs. DMSO or BMSCs-Control. LDH, lactate dehydrogenase; EdU, 5-Ethynyl-2′-deoxyuridine; TUNEL, terminal deoxynucleotidyl transferase-mediated dUTP nick-end labeling; GAPDH, glyceraldehyde-3-phosphate dehydrogenase; Bax, Bcl-2-associated X protein; Bcl-2, B-cell CLL/Lymphoma 2; IL-6, interleukin-6; IL-1β, interleukin-1β; TNF-α, tumor necrosis factor-α; BMSCs-control, astrocytes co-cultured with BMSCs-derived exosomes without any treatment; BMSCs-miR-NC, astrocytes co-cultured with BMSCs-derived exosomes infected with miR-NC; BMSCs-miR-138-5p, astrocytes co-cultured with BMSCs-derived exosomes infected with miR-138-5p overexpression; BMSCs, bone marrow-derived mesenchymal stem cells

An AChE activity test was performed, which revealed that the number of exosomes treated with GW4869 was significantly lower than that of those treated with DMSO (Fig. 7a). The results revealed that the expression of miR-138-5p in astrocytes treated with GW4869 was significantly lower than that in astrocytes treated with DMSO (Fig. 7b). Evidence was obtained indicating that the migration rate of the astrocytes co-cultured with BMSCs-miR-138-5p was higher than that of the astrocytes co-cultured with BMSCs-miR-NC or BMSCs-control (*p* < 0.05) (Fig. 7c). Moreover, LIVE/DEAD staining demonstrated that few of the astrocytes co-cultured with BMSCs-miR-138-5p were loose and presented with swelling, rupture or dissolution in varying degrees, accompanied with red-stained nucleus. The LDH release rate was the diminished in astrocytes co-cultured with BMSCs-miR-138-5p (Fig. 7d). EdU was employed to detect cell proliferation, which demonstrated that BMSCs-miR-138-5p led to the highest EDU positive rate which was accompanied by significantly increased cell proliferation ability (Fig. 7e). Cell apoptosis was detected by TUNEL staining, the results of which indicated that BMSCs-miR-138-5p resulted in a reduced apoptotic rate when compared with BMSCs-miR-NC and BMSCs-control (Fig. 7f). According to Western blot analysis, when compared with negative control co-culture, BMSCs-miR-138-5p co-culture decreased the expression of cleaved caspase-3 and Bax, accompanied by increased Bcl-2 (*p* < 0.05), with elevated expression of proliferation factors CDK-4, CyclinD1 and Cyclin E (*p* < 0.05; Fig. 7g). These results suggest that miR-138-5p, when delivered to astrocytes by BMSCs, can promote astrocyte proliferation and inhibit astrocyte apoptosis.

### BMSCs-derived exosomal miR-138-5p reduces neuron injury following IS in vivo

**Fig. 8:**
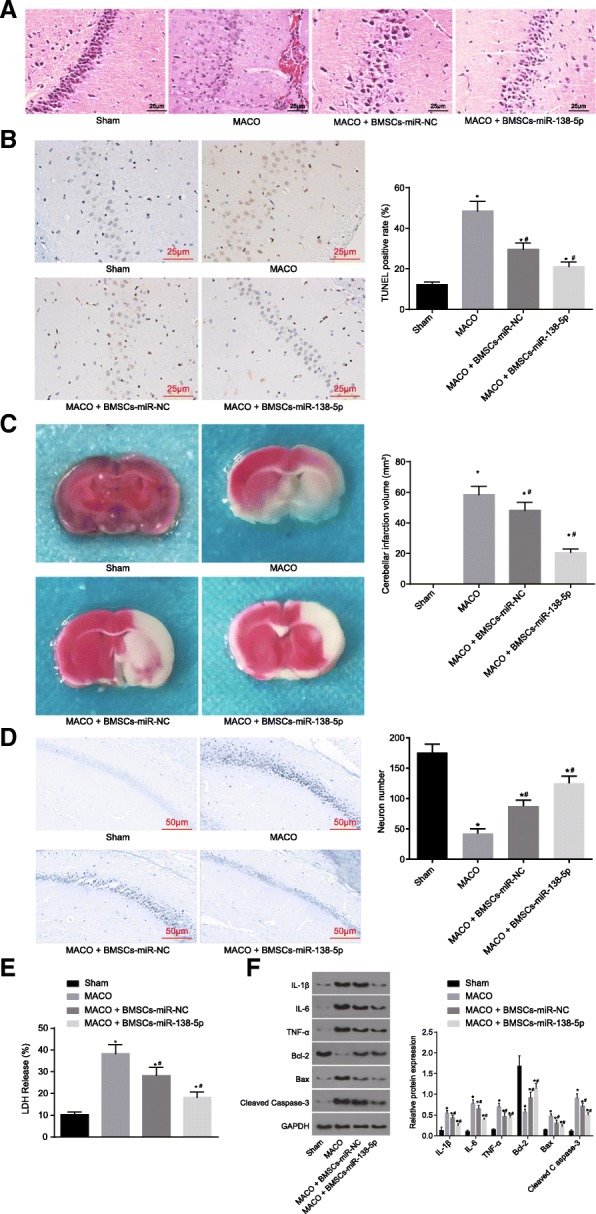
BMSCs-derived exosomal miR-138-5p reduces neuron injury following IS in vivo*.* **a**, HE staining for hippocampal tissues of MCAO mouse models (scale bar = 25 μm); **b**, TUNEL staining to detect neuron apoptosis (scale bar = 25 μm) and quantitative analysis for the apoptotic rate; **c**, TTC staining showing the volume changes of cerebral infarction in mice and the corresponding quantitative analysis; **d**, Nissl staining (scale bar = 50 μm) showing the number of neurons and the corresponding quantitative analysis; **e**, quantitative analysis for LDH content; **f**, protein expression of inflammatory factors, proliferation and apoptosis marker proteins determined by Western blot analysis. The data were all measurement data, expressed as mean ± standard deviation. The comparison among multiple groups was analyzed by one-way analysis of variance. The experiment was repeated three times. *, *p* < 0.05 vs. sham. #, *p* < 0.05 vs. MCAO. *n* = 10. LDH, lactate dehydrogenase; TUNEL, terminal deoxynucleotidyl transferase-mediated dUTP nick-end labeling; MCAO, middle cerebral artery occlusion; GAPDH, glyceraldehyde-3-phosphate dehydrogenase; Bax, Bcl-2-associated X protein; Bcl-2, B-cell CLL/Lymphoma 2; IL-6, interleukin-6; IL-1β, interleukin-1β; TNF-α, tumor necrosis factor-α; BMSCs-control, MCAO mice without any treatment; BMSCs-miR-NC, MCAO mice injected with BMSCs-miR-NC exosomes; BMSCs-miR-138-5p, MCAO mice injected with BMSCs-miR-138-5p exosomes

MCAO mouse models were established, followed by the application of HE staining to observe neuron injury among the IS mice. The volume of neurons was reduced, the intercellular space was widened, and the interstitial edema was more severe among the MCAO mice. However, among the MCAO mice treated with BMSCs-miR-NC or BMSCs-miR-138-5p brought about a significant degree of attenuation (Fig. 8a). Compared to the mice not subjected to any treatment, the MCAO mice exhibited an increased neuron apoptotic rate, cerebral infarction, and LDH content in the neurons, accompanied by reduced number of neurons. Relative to the MCAO mice, MCAO mice with treatments of BMSCs-miR-NC or BMSCs-miR-138-5p displayed a reduced neuron apoptotic rate, cerebral infarction, and LDH content in the neurons, as well as an increased number of neurons, while those with BMSCs-miR-138-5p exhibited a significant difference (Fig. 8b-e). The expression of inflammatory factors (IL-6, IL-1β, TNF-α) and apoptosis marker proteins (cleaved caspase-3, Bax and Bcl-2) were determined to evaluate inflammatory response and apoptosis (Fig. 8f). The results revealed that the expression of inflammatory factors (IL-6, IL-1β, TNF-α) and apoptosis marker proteins (cleaved caspase-3 and Bax) was higher in the MCAO mice, which was accompanied by lower Bcl-2 than those not subjected to any treatment. The presence of BMSCs-miR-NC or BMSCs-miR-138-5p treatment resulted in a significant decrease in IL-6, IL-1β, TNF-α and cleaved caspase-3 and Bax as well as increased Bcl-2, the difference of which was more significant following the administration of BMSCs-miR-138-5p. These results suggest that BMSCs-derived exosomal miR-138-5p could alleviate neuron injury in IS mice.

## Discussion

IS remains a chief contributor to adult death and disability worldwide [3]. Recent studies have highlighted the role of exosomal miRNAs in the process of important physiological functions, in addition to their potential as novel biomarkers and therapeutic targets for IS, owing to their influence on stroke severity, and treatment outcomes [22, 23]. In the present study, we aimed to explore the possible involvement of miR-138-5p and LCN2 in IS. The results obtained provided evidence that BMSCs-derived miR-138-5p-overexpressing exosomes promote proliferation while inhibiting the apoptosis of astrocytes in IS by targeting LCN2.

Initially, the results of the bioinformatics prediction followed by verification of dual luciferase reporter gene assay revealed that LCN2 is a target gene of miR-138-5p. In other diseases, miR-138-5p and LCN2 showed the regulatory relationship in consistent with our result. For example, downregulated miR-138 contributed to overexpressed LCN2 in colorectal cancer with liver metastasis [24]. Similarly, miR-138 was found to be able to negatively regulate LCN2 expression, which resulted in suppression of hypoxia-induced cardiomyocyte apoptosis [25]. Another key finding of this study is that LCN2 is highly expressed in IS and may affect the activities of OGD-induced astrocytes. LCN2 involves in multiple cellular processes such as innate immunity, apoptosis and renal development [26]. In concert with the results of the current study, LCN2 overexpression has been previously detected to enhance neuroinflammation in human OGD-induced astrocytes following IS [27]. Moreover, LCN2 expression was identified in the ischemic brain of individuals post temporary experimental ischemia with higher plasma levels of LCN2 detected among patients with IS patients, leading to poor overall clinical outcomes accompanied by post-stroke infections [28]. These data suggested that LCN2 might be a clinical indicator that indicated damage existed in brain tissue.

In addition, the overexpression of miR-138-5p was observed to stimulate the proliferation and migration of astrocytes injured as a result of hypoxia and glucose deprivation, coupled with inhibited apoptosis and inflammatory response, which were reversed by down-regulation of miR-138-5p. Furthermore, miR-138-5p not only plays a crucial role in the brain but also in other tissues. For example, He et al. concluded that miR-138 prevented hypoxia-induced apoptosis via the MLK3/JNK/c-jun pathway, ultimately conferring protection to cardiomyocytes [29]. Moreover, miR-138 has been shown to contribute to the inhibition of pulmonary artery smooth muscle cell apoptosis, suppression of caspases activation as well as disruption of Bcl-2 signaling in hypoxic pulmonary vascular remodeling by targeting Mst1 [30]. LCN2 secretion was activated during brain injuries and it was negatively regulated by miR-138-5p. A previous study demonstrated LCN2 secretion by brain astrocytes detected under inflammatory conditions and LCN2 promotes apoptosis, morphological changes, as well as migration in astrocytes both in vitro and in vivo [31], which is consistent with our results. Moreover, LCN2 can be secreted by astrocytes, which represents one of its main cellular sources under conditions of brain injury, thereby resulting in the activation of neuroinflammation [28]. In the present study, we set out to investigate the mechanism by which miR-138-5p in astrocytes influences IS, the results of which revealed that the overexpression of miR-138-5p could confer protection to astrocytes from injury induced by OGD via the downregulation of its target gene LCN2, which indicated miR-138-5p could be explored as a microRNA drug against IS.

Furthermore, evidence obtained indicated that BMSCs could deliver miR-138-5p to the astrocyte model through exosomes, with BMSCs-derived exosomal miR-138-5p exhibiting an ability to efficiently influence inflammation and neuronal injury in IS models in vivo. Exosomes are equipped with functional molecules and are capable of transferring information between cells and regulating multiple physiological and pathological processes [32]. Many cells from multiple tissues are able to secret exosomes to carry out physiological function, including that of red blood cells, dendritic cells and even tumor cells. As previously reported, astrocyte exosomes confer protection to hypoxic-ischemic neurons [18]. Various miRNAs have been reported to be loaded by exosomes that play roles in inflammation and neuron injury. For instance, BMSCs are capable of exerting therapeutic effects through exosomes. BMSCs-derived miR-223-containing exosomes have been identified to induce liver protection in experimental autoimmune hepatitis in part by reducing expression levels of inflammatory cytokines [33]. Moreover, exosomes from miR-30d-5p-adipose-derived stem cells have been shown to confer a protective effect on acute IS, ultimately attenuating autophagy-mediated brain injury via the promotion of M2 microglial/macrophage polarization [34]. In addition, exosomal miR-21 derived from H_2_O_2_-treated MSCs can be delivered to C-kit+ cardiac stem cells to downregulate PTEN expression, thus conferring protection against oxidative stress-triggered cell death in the ischemic myocardium via activation of the PI3K/AKT signaling [35]. Microglial exosomal miR-124-3p has been linked with the inhibition of neuronal inflammation in scratch-injured neurons following traumatic brain injury [36]. Although available cell therapy for neurological diseases such as stroke is still in clinical trials [37], strong evidence regarding cell-based treatment for stroke highlighted the progress of BMSCs-derived exosomes for stroke treatment [38]. In addition, the miRNAs in BMSCs-derived exosomes can be altered to expand the therapeutic effect of exosomes [39, 40].

In conclusion, this study demonstrates that BMSCs-derived exosomal miR-138-5p is capable of negatively regulating the expression of LCN2, under which circumstances it promotes proliferation while inhibiting apoptosis and inflammatory responses of astrocytes following IS, highlighting a novel treatment strategy using miRNAs loaded exosomes. However, there are still many unknowns about the study of exosomes in the field of stroke, including how the parenchymal cells or remote organs affect the secretion of exosomes and the endogenous substances through signaling pathways, and how the exosomes regulate the expression of endogenous genes in recipient cells. These questions are waiting for us to explore one by one.

## Data Availability

Data sharing not applicable to this article as no data-sets were generated or analyzed during the current study.

## Abbreviations

AI: Apoptotic index
ANOVA: Analysis of variance
BMSCs: Bone marrow-derived mesenchymal stem cells
BSA: Bovine serum albumin
CFSE: 5-(and − 6)-carboxyfluorescein diacetate succinimidyl ester
DMEM: Dulbecco’s modified Eagle’s Medium
DTNB: 5,5′-Dithiobis (2-nitrobenzoic acid)
ECL: Chemiluminescence
EdU: 6-Ethynyl-2′-deoxyuridine
FITC: Fluorescein isothiocyanate
GAPDH: Glyceraldehyde-3-phosphate dehydrogenase
GFAP: Glial fibrillary acidic protein
HE: Hematoxylin and eosin
HRP: Peroxidase horseradish
IgG: immunoglobulin G
IL-6: Interleukin-6
IS: Ischemic stroke
LCN2: Lipocalin 2
LDH: Lactate dehydrogenase
MCAO: Middle cerebral artery occlusion
miRNAs/miRs: microRNAs
MUT: Mutant type
NC: Negative control
OGD: Oxygen-glucose deprivation
PAGE: Polyacrylamide gel electrophoresis
PVDF: Polyvinylidene fluoride
TEM: Transmission electron microscopy
TTC: 2,3,5-triphenyltetrazolium chloride
TUNEL: Terminal deoxynucleotidyl transferase-mediated dUTP nick-end labeling
WT: Wild type

## References

[1] Marei HE, Hasan A, Rizzi R, Althani A, Afifi N, Cenciarelli C, Caceci T, Shuaib A (2018). Potential of stem cell-based therapy for ischemic stroke. Front Neurol.

[2] Donnan GA, Fisher M, Macleod M, Davis SM (2008). Stroke. Lancet.

[3] Gabryel B, Kasprowska D, Kost A, Labuzek K, Urbanek T (2015). Astrocytes in ischemic stroke - a potential target for neuroprotective strategies. Postepy Hig Med Dosw (Online).

[4] Jin R, Yang G, Li G (2010). Inflammatory mechanisms in ischemic stroke: role of inflammatory cells. J Leukoc Biol.

[5] Liu Z, Chopp M (2016). Astrocytes, therapeutic targets for neuroprotection and neurorestoration in ischemic stroke. Prog Neurobiol.

[6] Choudhury GR, Ding S (2016). Reactive astrocytes and therapeutic potential in focal ischemic stroke. Neurobiol Dis.

[7] Tian T, Zhang HX, He CP, Fan S, Zhu YL, Qi C, Huang NP, Xiao ZD, Lu ZH, Tannous BA, Gao J (2018). Surface functionalized exosomes as targeted drug delivery vehicles for cerebral ischemia therapy. Biomaterials.

[8] Shao Y, Shen Y, Chen T, Xu F, Chen X, Zheng S (2016). The functions and clinical applications of tumor-derived exosomes. Oncotarget.

[9] Wang Yan, Zhao Ranzun, Liu Debin, Deng Wenwen, Xu Guanxue, Liu Weiwei, Rong Jidong, Long Xianping, Ge Junbo, Shi Bei (2018). Exosomes Derived from miR-214-Enriched Bone Marrow-Derived Mesenchymal Stem Cells Regulate Oxidative Damage in Cardiac Stem Cells by Targeting CaMKII. Oxidative Medicine and Cellular Longevity.

[10] Deng M, Xiao H, Peng H, Yuan H, Xu Y, Zhang G, Tang J, Hu Z (2018). Preservation of neuronal functions by exosomes derived from different human neural cell types under ischemic conditions. Eur J Neurosci.

[11] Dong M, Xi G, Keep RF, Hua Y (2013). Role of iron in brain lipocalin 2 upregulation after intracerebral hemorrhage in rats. Brain Res.

[12] Bi F, Huang C, Tong J, Qiu G, Huang B, Wu Q, Li F, Xu Z, Bowser R, Xia XG, Zhou H (2013). Reactive astrocytes secrete lcn2 to promote neuron death. Proc Natl Acad Sci U S A.

[13] Zhang Z, Xu G, Cai B, Zhang H, Zhu W, Liu X (2017). Genetic variants in MicroRNAs predict recurrence of ischemic stroke. Mol Neurobiol.

[14] Tang XJ, Yang MH, Cao G, Lu JT, Luo J, Dai LJ, Huang KM, Zhang LI (2016). Protective effect of microRNA-138 against cerebral ischemia/reperfusion injury in rats. Exp Ther Med.

[15] Lugli G, Cohen AM, Bennett DA, Shah RC, Fields CJ, Hernandez AG, Smalheiser NR (2015). Plasma Exosomal miRNAs in persons with and without Alzheimer disease: altered expression and prospects for biomarkers. PLoS One.

[16] Song K, Huang M, Shi Q, Du T, Cao Y (2014). Cultivation and identification of rat bone marrow-derived mesenchymal stem cells. Mol Med Rep.

[17] Ayuk SM, Abrahamse H, Houreld NN (2016). The role of photobiomodulation on gene expression of cell adhesion molecules in diabetic wounded fibroblasts in vitro. J Photochem Photobiol B.

[18] Huang JL, Qu Y, Tang J, Zou R, Li SP, Li YF, Zhang L, Xia B, Mu DZ (2018). Zhongguo Dang Dai Er Ke Za Zhi.

[19] Xu B, Shao Q, Xie K, Zhang Y, Dong T, Xia Y, Tang W (2016). The Long non-coding RNA ENST00000537266 and ENST00000426615 influence papillary thyroid Cancer cell proliferation and motility. Cell Physiol Biochem.

[20] Wei H, Li Y, Han S, Liu S, Zhang N, Zhao L, Li S, Li J (2016). cPKCgamma-modulated autophagy in neurons alleviates ischemic injury in brain of mice with ischemic stroke through Akt-mTOR pathway. Transl Stroke Res.

[21] Suk K (2016). Lipocalin-2 as a therapeutic target for brain injury: an astrocentric perspective. Prog Neurobiol.

[22] Chen Y, Song Y, Huang J, Qu M, Zhang Y, Geng J, Zhang Z, Liu J, Yang GY (2017). Increased circulating Exosomal miRNA-223 is associated with acute ischemic stroke. Front Neurol.

[23] Li DB, Liu JL, Wang W, Li RY, Yu DJ, Lan XY, Li JP (2017). Plasma Exosomal miR-422a and miR-125b-2-3p serve as biomarkers for ischemic stroke. Curr Neurovasc Res.

[24] Cristobal I, Torrejon B, Gonzalez-Alonso P, Manso R, Rojo F, Garcia-Foncillas J (2016). Downregulation of miR-138 as a contributing mechanism to Lcn-2 overexpression in colorectal Cancer with liver metastasis. World J Surg.

[25] Xiong H, Luo T, He W, Xi D, Lu H, Li M, Liu J, Guo Z (2016). Up-regulation of miR-138 inhibits hypoxia-induced cardiomyocyte apoptosis via down-regulating lipocalin-2 expression. Exp Biol Med (Maywood).

[26] Yang J, Goetz D, Li JY, Wang W, Mori K, Setlik D, Du T, Erdjument-Bromage H, Tempst P, Strong R, Barasch J (2002). An iron delivery pathway mediated by a lipocalin. Mol Cell.

[27] Zhang Y, Liu J, Yang B, Zheng Y, Yao M, Sun M, Xu L, Lin C, Chang D, Tian F (2018). Ginkgo biloba extract inhibits Astrocytic Lipocalin-2 expression and alleviates Neuroinflammatory injury via the JAK2/STAT3 pathway after ischemic brain stroke. Front Pharmacol.

[28] Hochmeister S, Engel O, Adzemovic MZ, Pekar T, Kendlbacher P, Zeitelhofer M, Haindl M, Meisel A, Fazekas F, Seifert-Held T (2016). Lipocalin-2 as an infection-related biomarker to predict clinical outcome in ischemic stroke. PLoS One.

[29] He S, Liu P, Jian Z, Li J, Zhu Y, Feng Z, Xiao Y (2013). miR-138 protects cardiomyocytes from hypoxia-induced apoptosis via MLK3/JNK/c-Jun pathway. Biochem Biophys Res Commun.

[30] Li S, Ran Y, Zhang D, Chen J, Li S, Zhu D (2013). MicroRNA-138 plays a role in hypoxic pulmonary vascular remodelling by targeting Mst1. Biochem J.

[31] Lee S, Jha MK, Suk K (2015). Lipocalin-2 in the inflammatory activation of brain astrocytes. Crit Rev Immunol.

[32] Tian T, Zhu YL, Zhou YY, Liang GF, Wang YY, Hu FH, Xiao ZD (2014). Exosome uptake through clathrin-mediated endocytosis and macropinocytosis and mediating miR-21 delivery. J Biol Chem.

[33] Chen L, Lu FB, Chen DZ, Wu JL, Hu ED, Xu LM, Zheng MH, Li H, Huang Y, Jin XY, Gong YW, Lin Z, Wang XD, Chen YP (2018). BMSCs-derived miR-223-containing exosomes contribute to liver protection in experimental autoimmune hepatitis. Mol Immunol.

[34] Jiang M, Wang H, Jin M, Yang X, Ji H, Jiang Y, Zhang H, Wu F, Wu G, Lai X, Cai L, Hu R, Xu L, Li L (2018). Exosomes from MiR-30d-5p-ADSCs reverse acute ischemic stroke-induced, autophagy-mediated brain injury by promoting M2 microglial/macrophage polarization. Cell Physiol Biochem.

[35] Shi B, Wang Y, Zhao R, Long X, Deng W, Wang Z (2018). Bone marrow mesenchymal stem cell-derived exosomal miR-21 protects C-kit+ cardiac stem cells from oxidative injury through the PTEN/PI3K/Akt axis. PLoS One.

[36] Huang S, Ge X, Yu J, Han Z, Yin Z, Li Y, Chen F, Wang H, Zhang J, Lei P (2018). Increased miR-124-3p in microglial exosomes following traumatic brain injury inhibits neuronal inflammation and contributes to neurite outgrowth via their transfer into neurons. FASEB J.

[37] Zhou Y, Xu H, Xu W, Wang B, Wu H, Tao Y, Zhang B, Wang M, Mao F, Yan Y, Gao S, Gu H, Zhu W, Qian H (2013). Exosomes released by human umbilical cord mesenchymal stem cells protect against cisplatin-induced renal oxidative stress and apoptosis in vivo and in vitro. Stem Cell Res Ther.

[38] Hess DC, Borlongan CV (2008). Cell-based therapy in ischemic stroke. Expert Rev Neurother.

[39] Xin H, Li Y, Liu Z, Wang X, Shang X, Cui Y, Zhang ZG, Chopp M (2013). MiR-133b promotes neural plasticity and functional recovery after treatment of stroke with multipotent mesenchymal stromal cells in rats via transfer of exosome-enriched extracellular particles. Stem Cells.

[40] Xin H, Li Y, Buller B, Katakowski M, Zhang Y, Wang X, Shang X, Zhang ZG, Chopp M (2012). Exosome-mediated transfer of miR-133b from multipotent mesenchymal stromal cells to neural cells contributes to neurite outgrowth. Stem Cells.

