# Correction to: Exosomes derived from microRNA-138-5poverexpressing bone marrow-derived mesenchymal stem cells confer neuroprotection to astrocytes following ischemic stroke via inhibition of LCN2

**DOI:** 10.1186/s13036-022-00285-w

**Published:** 2022-03-31

**Authors:** Yiming Deng, Duanduan Chen, Feng Gao, Hong Lv, Guojun Zhang, Xuan Sun, Lian Liu, Dapeng Mo, Ning Ma, Ligang Song, Xiaochuan Huo, Tianyi Yan, Jingbo Zhang, Zhongrong Miao

**Affiliations:** 1 Department of Interventional Neuroradiology, Beijing Tiantan Hospital, Capital Medical University, No. 6, Tiantan Xili, Fengtai District, Beijing, 100050 People’s Republic of China; 2 China National Clinical Research Center for Neurological Diseases, Beijing, 100070 People’s Republic of China; 3 Center of Stroke, Beijing Institute for Brain Disorders, Beijing, 100069 China; 4 School of Life Science, Beijing Institute of Technology, Beijing, 100081 China; 5 Departments of Clinical Laboratory, Beijing Tiantan Hospital, Capital Medical University, Beijing, 100050 People’s Republic of China

**Correction to: J Biol Eng 13, 71 (2019)**

**https://doi.org/10.1186/s13036-019-0193-0**

Figure 8 of the original article [1] unfortunately contained an error: Image 8b that was used as a template during the preparation of the manuscript, was accidentally left in the final version of the article that went on to publish online.

The correct image presenting the TUNEL staining to detect neuron apoptosis and to facilitate quantitative analysis for the apoptotic rate, is included in Fig. 8 below as well as the original article which has now been revised.

**Fig. 8:**
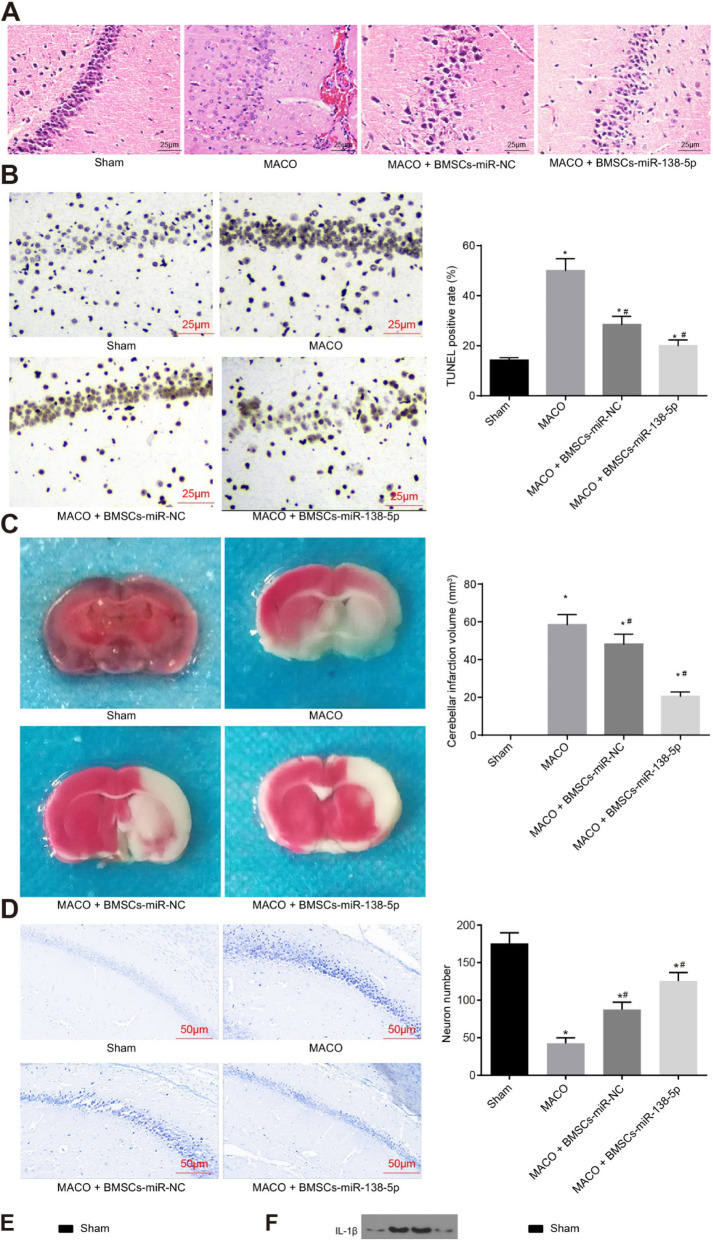
BMSCs-derived exosomal miR-138-5p reduces neuron injury following IS in vivo. **a**, HE staining for hippocampal tissues of MCAO mouse models (scale bar = 25 μm); **b**, TUNEL staining to detect neuron apoptosis (scale bar = 25 μm) and quantitative analysis for the apoptotic rate; **c**, TTC staining showing the volume changes of cerebral infarction in mice and the corresponding quantitative analysis; **d**, Nissl staining (scale bar = 50 μm) showing the number of neurons and the corresponding quantitative analysis; **e**, quantitative analysis for LDH content; **f**, protein expression of inflammatory factors, proliferation and apoptosis marker proteins determined by Western blot analysis. The data were all measurement data, expressed as mean ± standard deviation. The comparison among multiple groups was analyzed by one-way analysis of variance. The experiment was repeated three times. *, *p* < 0.05 vs. sham. #, *p* < 0.05 vs. MCAO. *n* = 10. LDH, lactate dehydrogenase; TUNEL, terminal deoxynucleotidyl transferase-mediated dUTP nick-end labeling; MCAO, middle cerebral artery occlusion; GAPDH, glyceraldehyde-3-phosphate dehydrogenase; Bax, Bcl-2-associated X protein; Bcl-2, B-cell CLL/Lymphoma 2; IL-6, interleukin-6; IL-1β, interleukin-1β; TNF-α, tumor necrosis factor-α; BMSCs-control, MCAO mice without any treatment; BMSCs-miR-NC, MCAO mice injected with BMSCs-miR-NC exosomes; BMSCs-miR-138-5p, MCAO mice injected with BMSCs-miR-138-5p exosomes
